# Genetic Diversity of Bacillus thuringiensis from Different Geo-Ecological Regions of Ukraine by Analyzing the 16S rRNA and gyrB Genes and by AP-PCR and saAFLP

**Published:** 2013

**Authors:** N. V. Punina, V. S. Zotov, A. L. Parkhomenko, T. U. Parkhomenko, A. F. Topunov

**Affiliations:** Bach Institute of Biochemistry, Russian Academy of Sciences, Leninsky prospect, 33, bld. 2, Moscow, Russia, 119071; Research Centre for Medical Genetics, Russian Academy of Medical Sciences, Moskvorechje str.,1, Moscow, Russia, 115478; Institute of Agriculture of Crimea, National Academy of Agrarian Sciences of Ukraine, Kievskaya str., 150, Simferopol, Ukraine, 95453

**Keywords:** Bacillus cereus group, B. thuringiensis, 16S ribosomal RNA, gyrB, saAFLP, taxonomy, phylogeny

## Abstract

The *Bacillus cereus *group consists of closely related species of bacteria and is of interest to researchers due to its importance in industry and medicine. However, it remains difficult to distinguish these bacteria at the intra- and inter-species level. *Bacillus thuringiensis (Bt)* is a member of the *B. cereus* group. In this work, we studied the inter-species structure of five entomopathogenic strains and 20 isolates of *Bt*, which were collected from different geo-ecological regions of Ukraine, using various methods: physiological and biochemical analyses, analysis of the nucleotide sequences of the 16S rRNA and *gyrB *genes, by AP-PCR (BOX and ERIC), and by saAFLP. The analysis of the 16S rRNA and *gyrB* genes revealed the existence of six subgroups within the*B.cereus* group: *B anthracis*, *B. cereus* I and II, *Bt* I and II, and* Bt *III, and confirmed that these isolates belong to the genus *Bacillus*. All strains were subdivided into 3 groups. Seventeen strains belong to the group *Bt *II of commercial, industrial strains. The AP-PCR (BOX and ERIC) and saAFLP results were in good agreement and with the results obtained for the 16S rRNA and *gyrB* genes. Based on the derived patterns, all strains were reliably combined into 5 groups. Interestingly, a specific pattern was revealed by the saAFLP analysis for the industrial strain *Bt* 0376 р.о., which is used to produce the entomopathogenic preparation “STAR-t”.

## INTRODUCTION

*Bacillus thuringiensis *(*Bt*) are gram-positive bacteria that exhibit bioinsecticide activity due to their ability to produce δ-endotoxins (ICPs), or Cry proteins, during sporulation [1]. These toxins are active for a wide range of insect species and genera, including agricultural pests and human parasites [2, 3]. Due to the high specificity of ICPs, entomopathogenic *Bt* bacteria can be used, instead of pesticides, and are widely employed in designing bioengineered crop protection agents [4, 5].

Based on a phenotypic and genotypic analysis,* Bt *species were attributed to the *B. cereus *group. This group also comprises the closely related species* B. cereus*, *B. anthracis*, *B. mycoides*, *B. pseudomycoides*, and *B. weihеnstephanensis*. The *B. сereus *and *Bt *species cannot be distinguished using the morphological [6], phenotypic [7], or genetic methods [8–11]. It has been hypothesized that these species can belong to the same species, *B. cereus sensu lato* [12, 13]. Since this group of closely related bacteria is of significant interest for agriculture and medicine, a thorough investigation into their taxonomy, as well as an elaboration of new tools and technologies for their differentiation and isolation, remains a rather urgent task.

*Bt *strains were conventionally isolated and further divided into subspecies according to either the presence or absence of ICP crystals or the genes encoding them (*cry *and *cty*) [1, 3]. However, this method has a drawback: the ICP’s genes are localized on the plasmid, and bacteria can lose them or pass them to the other *Bt* strains or closely related bacterial species during conjugation [14]. Over 82 *Bt *serovars were revealed by a serological analysis of the flagellar antigen (H-serotyping) [15, 16]. However, such classification did not always correlate with the actual phylogenetic relationships for this species [17–19].

The genetic diversity of *Bt *bacteria and the possibility to distinguish between the two species, *Bt *and *B. cereus*, were studied using different methods: DNADNA hybridization [20] and the analysis of the nucleotide sequences of 16S rRN A, 23S rRN A, 16S–23S rRN A [8, 11], MLST [21], MEE [12, 18], AFLP [22–24], RFLP [25], AP-PCR [26–29], etc. However, the actual phylogenetic relationships between *Bt *have not been determined by these methods.

This work was aimed at assessing how the modified genomic fingerprinting technique (saAFLP) could be applied to reveal the phylogenetic differences between* Bacillus *sp. isolates and strains from various geo-ecological regions of Ukraine. The nucleotide sequences of the 16S rRN A and *gyrB *genes were analyzed in order to determine the taxonomic relationships at the genus– species level. The saAFLP method, along with other informative methods (rep-PCR ), was used to study the structure at the intra-species level. This complex diagnostics, together with the results of physiological and biochemical assays, offers broad opportunities for studying the taxonomic structure of these closely related organisms. However, it should be borne in mind that the sampling of *Bt *strains requires further broadening.

## MATERIALS AND METHODS

**Bacterial strains**

Five entomopathogenic strains and 20 isolates of *Bt* bacteria exhibiting unique biochemical properties from a collection of useful microorganisms of various Ukrainian and Russian research institutions (Institute of Agriculture of Crimea, National Academy of Agrarian Sciences of Ukraine, Simferopol, Autonomous Republic of Crimea, Ukraine; Institute of Agricultural Microbiology, National Academy of Agrarian Sciences of Ukraine, Chernigov, Ukraine; All-Russian Collection of Industrial Microorganisms “GosNIIGenetika”, Moscow, Russia) were used in this study. Five strains from the collection of the All-Russian Collection of Industrial Microorganisms “GosNIIGenetika” were used as standard strains. Isolates from the collection of the Institute of Agriculture of the Crimea, National Academy of Agrarian Sciences of Ukraine, were isolated in different geo-ecological regions of Ukraine.

**DNA isolation**

The overall cellular DNA specimens were isolated from strains cultured on agarized TY medium (g/l): yeast extract – 1.0; peptone – 10.0; CaCl_2_ – 0.4; agar – 20.0. DNA was isolated from fresh cultures on days 1–2 of growth via sorption onto magnetic particles (Mini-prep kit, Silex, Russia).

**Phenotypic characterization**

The morphological and physiological-biochemical characteristics of the pure bacterial cultures were determined based on the general strategy of phenotypic differentiation described in *A Guide for Bacterial Identification* [30] and *Methods for General Bacteriology* [31].

**PCR amplification and sequencing of the 16S rRNA gene**

The PCR analysis and subsequent determination of the nucleotide sequences of the 16S rRN A gene [32] were conducted on a genetic analyzer using the universal primers 27f (5’-GTTT GATC MTGGCTC AG-3’) and 1492R (5’-TACGGYTACCTT GTT ACGACTT -3’) [33]. The amplified fragments were detected by electrophoresis in 1.5% agarose gel. Sequencing was carried out on a Genetic Analyzer 3130xl ABI automated sequencing machine (Applied Biosystems, USA).

**PCR amplification and sequencing of the** *gyrB* **gene**

The *gyrB* gene was amplified and sequenced using the previously constructed primer systems UP1 and UP2r [34], the *Bacillus *genus-specific primers designed by us, gyrB_F (5’-CTT GAAGGACT AGARGCAGT-3’) + gyrB_Rf (5’-CCTTC ACGAACATC YTC ACC -3’) and gyrB_Fr (5’-GGTGARGATGTTC GTGAAGG-3’) + gyrB_R (5’-TGGATAAAGTT ACGACGYGG-3’), and the protocol. The temperature-time profile of the reaction was as follows: the initial denaturation at 94^о^С – 2 min; then, 30 cycles: 94^о^С – 30 s, 62^о^С – 30 s, 72^о^С – 1 min; final elongation – 5 min at 72^о^С. The amplified fragments were revealed by electrophoresis in 1.5% agarose gel. Sequencing was carried out on a Genetic Analyzer 3130xl ABI (Applied Biosystems, USA).

**PCR using primers to different repeating elements (rep-PCR)**

The previously described primer systems [26, 27] ER IC1R 5’-ATGTAAGCTCCT GGGGATTC AC-3’; ER IC2 5’-AAGTAAGTGACT GGGGTGAGCG-3’; and BOXA1R 5’-CT ACGGCAAGGCGACGCT GACG-3’ were used for rep-PCR .

Amplification was carried out in 25 μl of the following mixture: 1X polymerase buffer BioTaq (17 mM (NH_4_)_2_SO_4_, 6 mM Tris-HCl, pH 8.8, 2 mM MgCl_2_), 5 nM dNT P, 50 ng of DNA template, 12.5 pM of the primer, and 1.25 AU of BioTaq DNA polymerase (Dialat Ltd., Russia). The temperature-time profile of the reaction: first cycle – 94^о^С, 2 min; subsequent 40 cycles – 94^о^С, 20 s; 40^о^С, 30 s and 72^о^С, 90 s; final elongation – 7 min at 72^о^С. The PCR products were analyzed by electrophoresis in a 1.5% agarose gel stained with ethidium bromide at a field intensity of 6 V/cm and documented using the BioDoc Analyze system (Biometra, Germany).

**saAFLP analysis [35]**

We had modified the AFLP method developed and patented by M. Zabeau and P. Vos [36]; its suitability for the analysis of closely related *Bt *strains was assessed in this study. The phylogenetic relationships between closely related strains of various species belonging to the genus *Rhizobium *had been successfully analyzed using this modified saAFLP method [35]. The saAFLP procedure comprises three steps: (i) simultaneous treatment of the extracted bacterial DNA in the same tubes using one of the restriction endonucleases (XmaJI, XbaI, PstI) and ligation with a singlestranded adapter Ad.CT AG1; (ii ) PCR amplification with a single primer complementary to the Ad.CT AG1 sequence; (iii ) electrophoretic separation of the PCR products in agarose gel. The fundamentally new aspects for this saAFLP method include conducting the restriction analysis and the ligase reaction in the same tube, using restriction endonucleases XmaJI (XbaI, PstI) to study the phylogenetic relationships between the *Bt *strains isolated in various geo-ecological regions of Ukraine, and using only the single-stranded adapter Ad.CT AG1.

The restriction analysis was carried out simultaneously with the ligation in 10 μl of the mixture containing 80 ng of the DNA sample, the ligase buffer (Fermentas, USA), 10 pM of the single-stranded adapter Ad.CT AG1 (5’-ctagCT GGAATC GATTCC AG-3’), 5 AU of T4 DNA ligase (Fermentas, USA), and 1 AU of restrictase XmaJI (XbaI, PstI). The resulting mixture was incubated at 37^о^С for 2 h. The reaction volume was then brought up to 100 μl. PCR was carried out on a Mastercycler Gradient Eppendorf amplifier in 25 μl of the mixture containing 1 X PCR buffer, 2.8 mM MgCl_2_, 0.2 mM dNT P, 2 μl of the restrictase–ligase mixture as a DNA template, 0.4 μl of primer Pr.CT AG1 (5’-CT GGAATC GATTCC AGctag-3’) complementary to the adapter, and 1 AU BioTaq DNA polymerase (Dialat Ltd., Russia). PCR amplification was carried out in the following mode: initial denaturation – 94^о^С, 2 min, followed by 30 cycles – 94^о^С, 30 s; 40^о^С, 30 s; 72^о^С, 3 min; final elongation – 5 min at 72^о^С.

**Analysis of nucleotide sequences**

The primary comparative analysis of the nucleotide sequences determined in this study and represented in the GenBank database was carried out using the NC BI Blast software [37]. Sequence alignment was performed using the CLUSTALW 1.75v. software [38]; the sequences were verified and edited using BioEdit 7.0.5.3 [39] and Mega 3.1 [40] editors. The phylogenetic trees were constructed in the Mega 3.1 software [40] using the neighbor joining (NJ) [41] and minimum evolution (ME) [42] methods. The statistical significance of the branching order of the resulting trees was determined using the bootstrap analysis by constructing 1,000 alternative trees.

## RESULTS AND DISCUSSION

**Analysis of the nucleotide sequences of the 16S rRNA gene**

**Fig. 1:**
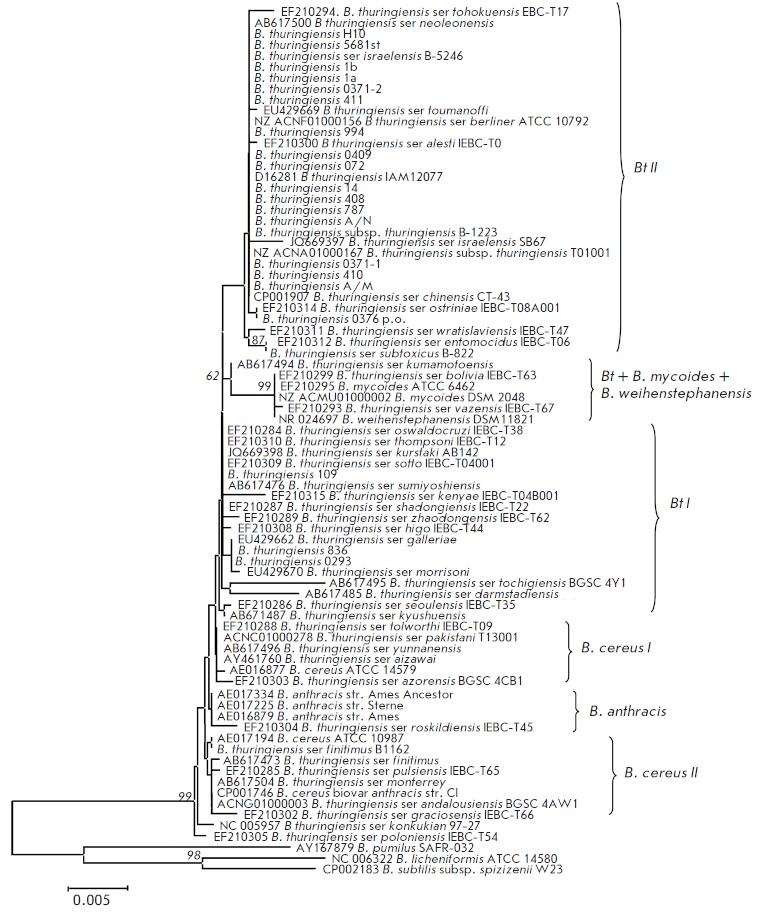
Phylogenetic tree constructed based on the sequences of the 16S rRNA gene for bacteria of the B. cereus group using the ME algorithm. The scale corresponds to 0.5 substitutions per 100 bp (genetic distances). The bootstrap confidence values were generated using 1,000 permutations and showed in % under the branches. Branches absent in more than 50% of the trees are not shown.

The analysis of the nucleotide sequences of the 16S rRN A gene is frequently used for taxonomic localization and the identification of the bacterial genus/species. We amplified and sequenced the PCR fragments of the 16S rRN A gene (the size of the sequenced region was 1386 bp) of five typical strains of genus *Bacillus *and 20 Ukranian isolates to verify their taxonomic attribution to the genus *Bacillus*. Similar nucleotide sequences of the 16S rRN A gene of *B. cereus*, *Bt*, *B. anthracis*, *B. mycoides*,* B. pseudomycoides*, and *B. weihеnstephanensis* were obtained from the database of the National Center for Biotechnology Information (NC BI, USA) and used for comparative purposes. The nucleotide sequences of *B. pumilus*, *B. licheniformis*, and *B. subtilis *were selected as the remote control for the phylogenetic analysis. A phylogenetic tree representing the evolution of the analyzed gene was constructed based on the aligned sequences using the ME algorithm (*Fig. 1*). The pairwise genetic distances were calculated using the Kimura’s two-parameter model.

The topology of the resulting tree was consistent with the phylogenetic structure of the genus determined by DNA–DNA hybridization [43] and established for the *B. сereus* group by the analysis of the 16S rRN A, 23S rRN A gene fragments [8, 11], and the 16S–23S rRN A intergenic region [44], rep-PCR [29], and AFLP [23].

The attribution of the isolates to the genus *Bacillus* has been verified by analysing the nucleotide sequences of the 16S rRN A gene. However, this method did not allow one to reliably distinguish individual species within the *B. cereus *group due to the fact that the sequence of the 16S rRN A gene was highly conserved (99.7–100.0% homology), which has also been repeatedly mentioned in other studies [8, 29].

The *B. anthracis *strains were grouped into a single cluster; however, the level of significance was low. *Bt* strains were also attributed to this cluster *B. anthracis*. We distinguished two *B. cereus *groups (I and II), identically to the study by Bavykin *et al. *[45]. This branching has not been statistically confirmed (statistical significance of the branching order < 50%). The *B. cereus *I group included the pathogenic *B. cereus *strain ATCC 14579^Т^ and a number of nonpathogenic *Bt *serovars. The* B. cereus *II group consisted of various *Bt* serovars and nonpathogenic *B. cereus* strain ATCC 10987^Т^. Most of the *Bt *strains with a low significance level of branching formed a single cluster, which brought together different serovars of this species. The *B. mycoides *and *B. weihenstephanensis *strains were attributed to a separate subgroup.

The potential commercial strains and the typical strain *Bt* ser. *berliner *ATCC 10792^Т^ were put together and attributed to the *Bt *II group with a branching significance of 56%. This group comprised seventeen Ukrainian isolates of different serotypes isolated from different host insects, mostly from the Lugansk and Kherson regions, and the Krasnogvardeisk and Simferopol districts. Strain *Bt* 0376 р.о. (serotype 1) was proposed for the production of the eco-friendly entomopathogenic preparation “STAR-t” (OOO Simbitor) intended to control the number of Colorado potato beetle (*Leptinotarsa decemlineata*) larvae, potato tuber moth (*Phtorimea operculella Zel*.), and chickpea leafminer (*Liriomiza cicerina Rd.*) during vegetation and storing potato and chickpea and was attributed to this group and had the group-specific substitutions A/G77, T/C90, T/A92, C/T192, C/A1015 in the 16S rRN A gene. All the investigated isolates from the *Bt *II group had completely identical nucleotide sequences of the 16S rRN A gene. Strain *Bt *var. *thuringiensis *994 (serotype 1, analogue of the bioagent of bacterial preparation “Bitoxybacillin”) used to produce the preparation “Akbitur,” strain *Bt *408 (serotype 3) exhibiting high entomopathogenic activity against *L. decemlineata*, and strain *Bt *var. *darmstadiensis *Н10 (serotype X) were also attributed to the *Bt *II group.

Strains *Bt *836 (serotype 4), *Bt *var. *kurstaki *0293 (serotype 3, analogue of the strain used as a bioagent in the preparation “Lepidocid”), and *Bt *var. *morrisoni* 109 (serotype X) were attributed to the *Bt* I group. Both specific nucleotide substitutions typical and unique for the *Bt *strains were found within each group. A total of 16, 30, 32, 28, and 21 substitutions were found in *B. anthracis, B. cereus *I, *B. cereus *II, *Bt* I, and *Bt *II, respectively. However, it should be mentioned that most nucleotide substitutions were random and strain-specific.

Thus, the 16S rRN A gene cannot be used to assess and study the phylogenetic relationships of the *B. cereus *group at a levels below genus/species, since it does not allow one to determine the species-specific nucleotide substitutions for this group.

**Genetic diversity of the sequences of the** *gyrB* **gene**

**Fig. 2:**
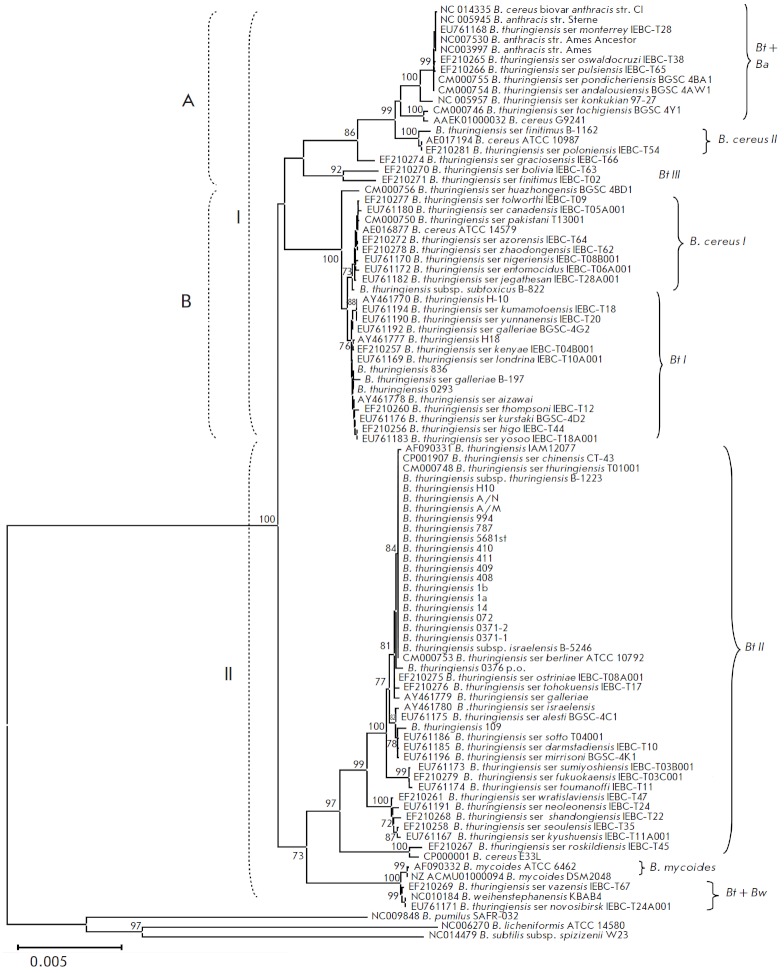
Phylogenetic tree constructed based on the sequences of the gyrB rRNA gene for bacteria of the B. cereus group using the ME algorithm. The scale corresponds to 5 substitutions per 100 bp (genetic distances). The bootstrap confidence values were generated using 1,000 permutations and showed in % under the branches. Branches absent in more than 50% of trees are not shown.

The nucleotide sequence of the *gyrB *gene is used along with the 16S rRN A gene in taxonomic studies and for bacterial identification [34]. A number of studies have recently been published where the variability of the sequence of this gene in different bacterial species belonging to the genus *Bacillus *was studied (e.g., *B. subtilis* [46], *B. cereus *groups [47]). The universal primers proposed earlier [34] and the primer systems constructed by us and specific for the 3’-terminus of the *gyrB* gene of bacteria belonging to the *B. cereus *group were used to amplify and sequence the PCR fragments of this gene (the size of the sequenced region was 1800 bp, 81.82% of the entire gene). We selected this fragment of the gene on the basis of the distribution of the polymorphism (entropy) level of the *gyrB *nucleotide sequence using the DNAsp v. 5 software [48]. The level of polymorphism was above average on the regions 150–700 and 1650–200 bp from the beginning of the gene (data not shown). However, due to the fact that there was a limited number of *gyrB *DNA sequences of a certain length of strains belonging to the *B. cereus* group in GenBank, we selected the region from 385 to 1507 bp from the beginning of the gene (the annotation is provided for the strain *Bt *ser. *berliner *ATCC 10792^Т^), which comprised 60% of the total length of the gene, for the analysis. The phylogenetic tree shown in *Fig. 2* was constructed using the ME algorithm for 25 investigated strains, isolates, and reference sequences of the *Bacillus* sp. strains included in GenBank. The nucleotide sequences of species *B. pumilus*, *B. licheniformis*, and *B. subtilis *were used as remote controls for the phylogenetic analysis.

The topology of the constructed tree was similar with that of the phylogenetic trees constructed earlier for the 16S rRN A gene, and for the intergenic region 16S–23S rRN A; it showed no dependence on the algorithms used for the construction (NJ, ME). The inter- and intraspecies differences between the species* B. anthracis *and the *B. cereus *– *Bt *group have been identified. The results of the studies demonstrated that the nucleotide sequence of the *gyrB *gene possesses a higher resolving power than the 16S rRN A gene and the intergenic region 16S–23S rRN A sequences [34, 46] and, hence, is more suitable for the taxonomic studies of closely related species.

**Fig. 3:**
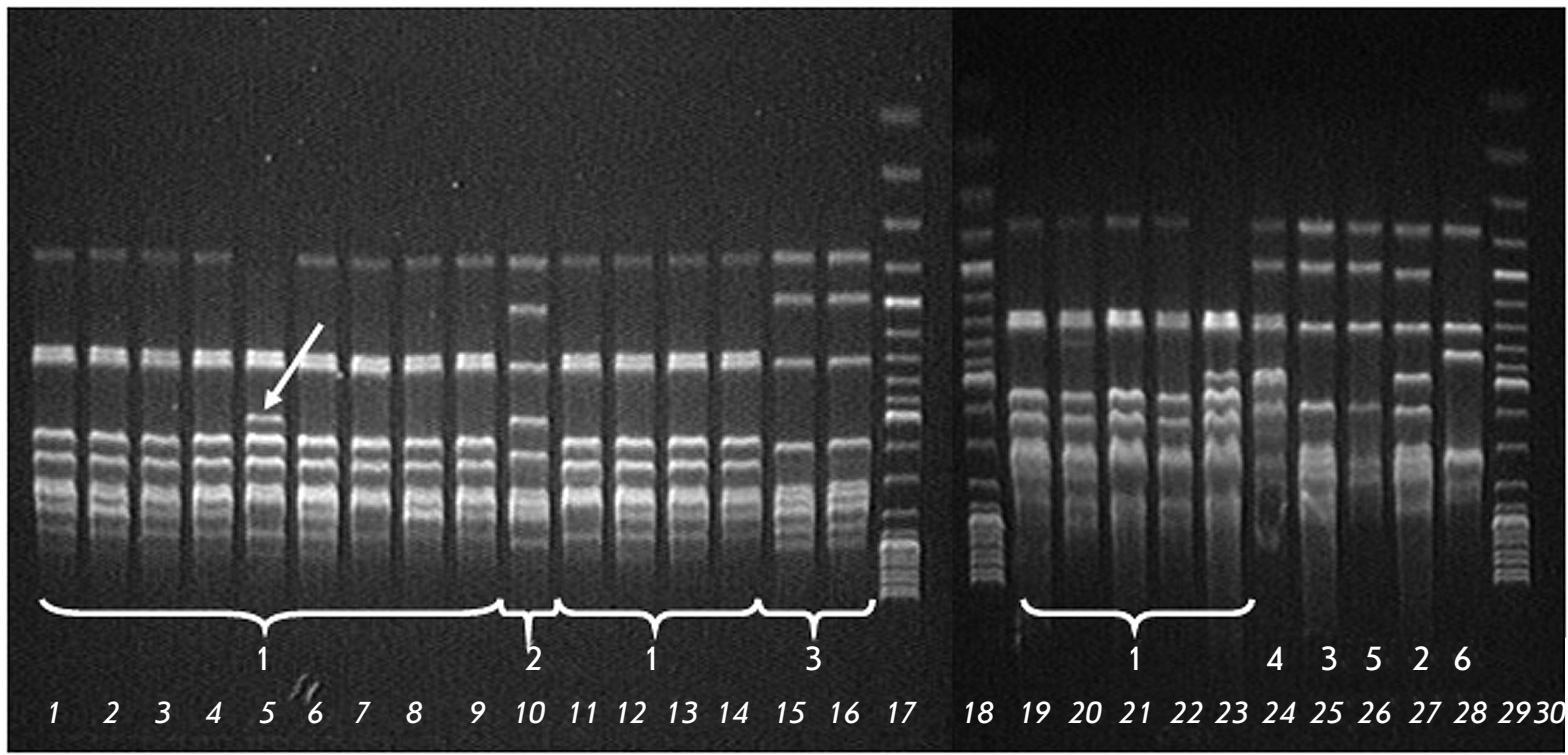
Electrophoretic analysis of the saAFLP products obtained for the DNA samples of *B. thuringiensis *strains. Lanes:** 17, 18, 29 **– 1 kb GeneRuler™ DNA molecular mass marker (Fermentas); **1 **– *Bt *Н10, R-type; **2 **– *Bt *A/N; **3 **– *Bt *408;** 4,19 **- *Bt *5681st; **5, 23 **– *Bt *0376 p.o.; **6 **– *Bt *787; **7 **– *Bt *411; **8 **– *Bt *72; **9 **– *Bt *0371-1; **10, 27 **– *Bt *109; **11 **– *Bt *14; **12** – *Bt *994; **13 **– *Bt *1b; **14 **- *Bt *A/M; **15 **– *Bt *836; **16, 25 **– *Bt *0293; **20 **– *Bt *sbsp. *israelensis *B-5246; **21 **– *Bt *0371; **22** – *Bt *sbsp. *thuringiensis *B-1223; **24 **– *Bt *sbsp. *subtoxicus *B-822; **26 **– *Bt *sbsp. *galeriae *B-197; **28 **– *Bt *sbsp. *finitimus* B-1162; **30 **– control PCR without template DNA.

Identically to the data obtained for the 16S rRN A gene (but with a higher significance level), five subgroups can be distinguished within the *B. cereus* group: *B. anthracis, B. cereus* I and II*, Bt *I and II. Another group, *Bt *III, was distinguished in the phylogenetic cladogram with a 92% statistical significance of the branching order. It comprised the following strains: *Bt* ser. *bolivia *IEBC-T63 and *Bt *ser. *finitimus *IEBC-T02. With a high significance level, most strains formed the *Bt *II group, which also comprised the strains used for the production of entomopathogenic preparations. However, as previously assumed based on published data, the *B. cereus *and *Bt *species were indistinguishable [45]. Thus, the *Bt *strains, along with the strains belonging to the species *B. anthracis *, *B. сereus, *and *B. weihenstephanensis *, were grouped into the subgroups* B. anthracis *, *B. cereus *I and II and *B. weihenstephanensis *. The *gyrB *gene of strain *Bt *0376 р.о. and other strains of this group were compared; due to the higher resolving power and variability of the *gyrB *nucleotide sequence, two substitutions specific to this strain (A/G861 and A/G1149) have been identified. In general, the level of similarity between the nucleotide and amino acid sequences in the *B. сereus *group was 87.1– 95.2% and 95.1–99.2%, respectively.

Clusterization of the strains into two groups with a high statistical significance of the branching order is worth mentioning. Cluster I was formed by groups A and B. Group A consisted of the reliably grouped pathogenic strains *B. anthracis, *the nonpathogenic strain B. cereus ATCC 10987^Т^, entomopathogenic strains *Bt* ser. *finitimus *B1162 and *Bt *ser. *poloniensis *IEBC-T54 belonging to the *B. cereus *II group, and entomopathogenic strains belonging to the *Bt *III group. Group B was formed by strain *B. cereus *ATCC 14579^Т^ (pathogenic for humans), entomopathogenic strains *Bt *belonging to the *B. cereus *I group, and entomopathogenic strains belonging to the *Bt *I group. Cluster II included bacteria belonging to the species *B. weihenstephanensis *and *B. mycoides*, and the *Bt *II group comprising most of the strains used for industrial production of entomopathogenic preparations. This clusterization of bacteria probably attests to a paraphyletic structure of both the *B. cereus *group in general and the separate species of this group.

**Polymorphism among** *Bt* **detected using saAFLP and rep-PCR markers**

**Fig. 4:**
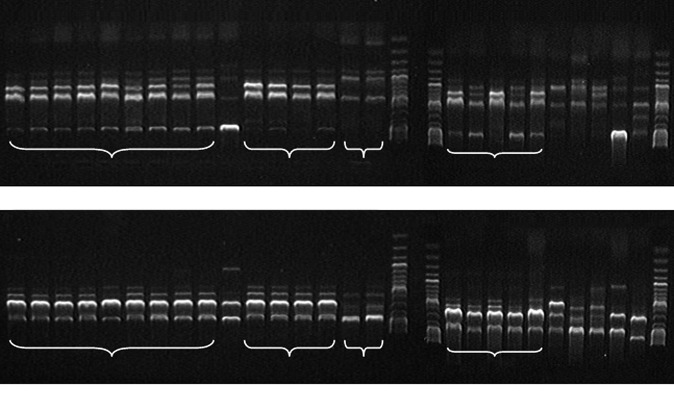
Electrophoretic analysis of the PCR products obtained for DNA samples of *B. thuringiensis *strains with primers ERIC (A) and BOX (B). Lanes: **17, 19, 30 **– 1 kb GeneRuler™ DNA molecular mass marker (Fermentas); **1 **– *Bt *Н10, Rtype;** 2 **– *Bt *A/N; **3 **– *Bt *408; **4,19 **– *Bt *5681st; **5, 23 **– *Bt *0376 p.o.; **6 **– *Bt *787; **7 **– *Bt *411; **8 **– *Bt *72; **9 **– *Bt *0371-1; **10, 28 **– *Bt *109; **11 **– *Bt *14; **12 **– *Bt *994; **13 **– *Bt *1b; **14 **– *Bt *A/M; **15 **– *Bt *836; **16, 26 **– *Bt *0293; **18 **– control PCR without template DNA; **21 **– *Bt *sbsp. *israelensis *B-5246; **22 **– *Bt *0371; **22 **– *Bt *sbsp. *thuringiensis *B-1223; **25 **– *Bt *sbsp. *subtoxicus* B-822; **27 **– *Bt *sbsp. *galeriae *B-197; **29 **– *Bt *sbsp. *finitimus *B-1162

Along with the housekeeping genes, genomic fingerprinting methods are used to reveal the differences between closely related bacterial species and strains. Rep- PCR is the most frequently used. This method is based on using oligonucleotide primers homologous to the sequences of various intergenic repeats. In our study, the differences between the closely related *Bt *strains were identified using rep-PCR (BOX-, ER IC-PCR ) and saAFLP. The results obtained are shown in *Figs. 3,4*.

All the strains under study were analyzed by saAFLP applying three restriction endonucleases (XmaJI, XbaI, and PstI). The informative spectra for all the *Bt *strains were recorded using XmaJI only. The modified saAFLP method allowed to distinguish the strains at the species–group level. All investigated *Bt* strains were divided into six group according to these spectra (*Fig. 3*). All strains presumably belonged to different subspecies of the *Bt *species. Group 1 comprised the typical strains *Bt *subsp*. thuringiensis *and *Bt *0376 p.o. This strain contained a unique saAFLP pattern (1,000 bp long), which distinguished it from the other strains belonging to group 1 (marked with a white arrow in *Fig. 3*). Groups 2, 3, 4, and 5 were represented by either a small number of strains or a single strain. It should be mentioned that this grouping corresponded to the data obtained previously by the analysis of 16S rRN A and *gyrB *genes sequences. It is significant that each strain of the same group was characterized by a group-specific saAFLP spectrum and pattern, which distinguished it from the strains belonging to the other groups. We also found patterns (markers) that are unique for individual strains (e.g., the commercial strain* Bt *0376 p.o.), which distinguished them among all the strains belonging to group 1. Thus, the proposed method is more specific and can be used for a quick search for strain/group-unique markers and for the study of polymorphism in populations.

The ER IC-PCR (*Fig. 4A*) and BOX-PCR (*Fig. 4B*) methods were also used in this study to compare the results obtained using these reference primers and by saAFLP analysis modified by us. Based on the analysis of the obtained ER IC and BOX patterns, all investigated* Bt *strains were subdivided into six groups. However, no differences between the ER IC and BOX spectra detected within each group for every strain. The number of specific PCR markers and the total number of fragments obtained by saAFLP were greater than those obtained using the ER IC and BOX primers. This fact attests to the higher sensitivity, specificity, and informativity of the saAFLP method. The character of the results could be attributed to the fact that the ER IC and BOX-PCR allow one to analyze only separate genomic regions, which are rather conserved (promoter regions (ER IC1R-ER IC2, BOX, RE P2-I-RE P1R-I) or the regions of functional genes (e.g., tRN A)). Hence, the spectra obtained by these methods contain general, rather than strain-specific, information and could be more useful for passportization of strains. The spectra recorded by saAFLP, which is not confined to any particular genomic region, show the individuality of each microorganism. The differences between all the analyzed strains could be identified on the basis of fingerprints (patterns) based on this method [34]. The specificity of the spectra allows one to conclude that the saAFLP method is probably appropriate for investigating and distinguishing the true phylogenetic relationships between bacteria without using the data obtained through other primers or methods, with the exception of determining the genus of a microorganism using the 16S rRN A or *gyrB *gene.

**Fig. 5:**
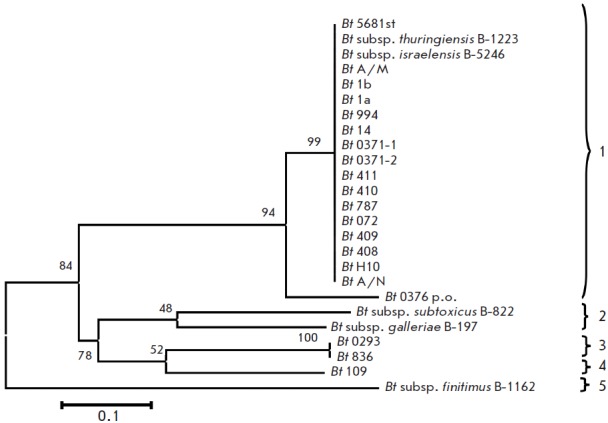
Dendrogram of 25 *Bt* strains isolated on the territory of Ukraine. The dendrogram was constructed using the NJ algorithm based on the total results of ERIC and BOX PCR and saAFLP for 36 polymorphic markers. The numbers denote the statistical significance of the branching order (%) determined by a bootstrap analysis of 1,000 permutations

However, in order to verify the reliability of these results and obtain a complete view of the genetic relationships between closely related bacteria, it is necessary to analyze the overall data obtained by both the saAFLP method and ER IC- and BOX-PCR . In our study, we used three methods (ER IC-, BOXPCR , and saAFLP) to identify 36 polymorphic markers (unique fragments) among the analyzed strains. The resulting data were used to construct a dendrogram (*Fig. 5*). The genetic distances between the strain pairs were determined using Pearson’s correlation, the Simple difference, and the Cosine distance (data not shown). The resulting matrix distances were used to conduct a cluster analysis using the NJ method. According to the results obtained, all the investigated strains were subdivided into five clusters. The statistical significance of the branching order varied from 58 to 99%. The clusters were isolated based on a similarity of ≥80% and/or a significance level of branching ≥50%. Cluster 1 comprised the strains *Bt *Н10 R-type, *Bt *A/N,* Bt *408, *Bt *409, *Bt *410, *Bt *5681st, *Bt *787, *Bt *411, *Bt *072,* Bt *0371-1, *Bt *14, *Bt *994, *Bt *1а, *Bt *1b, *Bt *A/M, *Bt *subsp*. israelensis *B-5246, *Bt *0371-1, *Bt *0371-2, and the typical strain *Bt *subsp*. thuringiensis *B-1223. Within this cluster, the strain *Bt *0376 р.о. was isolated with a high statistical significance of the branching order. Cluster 2 was formed by two subspecies, *Bt *subsp. *galleriae* and *Bt *subsp*. subtoxicus*. A significance of branching of <50% demonstrates that these strains presumably belong to two different subspecies and may represent separate clusters if the strain sampling is enlarged. In order to verify or refute this hypothesis, the strain sample should be broadened. Cluster 3 consisted of the strains *Bt *0293 and *Bt *836; clusters 4 and 5 were represented by the strains *Bt *109 and *Bt *subsp. *finitimus* B-1162, respectively.

Based on the published data and the results obtained by us, it can be concluded that a complex approach combining an analysis of both the biochemical properties of the strain and molecular-biological methods, is required to study and identify the *Bt *species belonging to the *B. сereus *group. The *Bt *species can be successfully studied using the nucleotide sequence of the* gyrB *gene. At the intraspecies level, it can be studied by saAFLP, along with the other AP-PCR methods (rep-PCR ). These methods were used to subdivide the strain sample into five groups, which also corresponded to their unique biochemical properties that had previously been determined in studies conducted by our colleagues [49]. The elaborated saAFLP method enabled to identify the DNA fragment, which is unique for the strain *Bt *0376 p.o. ., isolated first by our colleagues from the Institute of Agriculture of the Crimea (National Academy of Agrarian Sciences of Ukraine) and used to produce the entomopathogenic preparation “STAR-t”. We intend to increase the size of the strain sampling, to study the composition of the *cry *genes, and to determine the nucleotide sequences of the unique DNA fragments revealed for the separate saAFLP groups and strains.

## Abbreviations

Bt: Bacillus thuringiensis
ICPs: δ-endotoxins
b.p.: base pair
MLST: multilocus sequence typing
MEE: multilocus enzyme electrophoresis
AFLP: amplified fragment length polymorphism
saAFLP: single adapter amplified fragment length polymorphism
RFLP: restriction fragment length polymorphism
AP-PCR: arbitrarily primed polymerase chain reaction
rep-PCR: repetitive sequence-based PCR
BOX: DNA repeat
ERIC: DNA repeat
ME: minimum evolution
NJ: neighbor joining
